# Comparative Study of Hematopoietic Differentiation between Human Embryonic Stem Cell Lines

**DOI:** 10.1371/journal.pone.0019854

**Published:** 2011-05-16

**Authors:** Heather Melichar, Ou Li, Jenny Ross, Hilary Haber, Dragana Cado, Hector Nolla, Ellen A. Robey, Astar Winoto

**Affiliations:** Division of Immunology and Pathogenesis, Department of Molecular and Cell Biology, University of California, Berkeley, California, United States of America; University of California, San Francisco, United States of America

## Abstract

Directed differentiation of human embryonic stem cells (hESCs) into any desired cell type has been hailed as a therapeutic promise to cure many human diseases. However, substantial roadblocks still exist for *in vitro* differentiation of hESCs into distinct cell types, including T lymphocytes. Here we examined the hematopoietic differentiation potential of six different hESC lines. We compare their ability to develop into CD34^+^ or CD34^+^CD45^+^ hematopoietic precursor populations under several differentiation conditions. Comparison of lymphoid potential of hESC derived- and fetal tissue derived-hematopoietic precursors was also made. We found diverse hematopoietic potential between hESC lines depending on the culture or passage conditions. In contrast to fetal-derived hematopoietic precursors, none of the CD34^+^ precursors differentiated from hESCs were able to develop further into T cells. These data underscore the difficulties in the current strategy of hESC forward differentiation and highlight distinct differences between CD34^+^ hematopoietic precursors generated *in vitro* versus *in vivo*.

## Introduction

Directed differentiation of human embryonic stem cells (hESCs) into a variety of cell types has vast promise in the context of personalized human therapeutics and also towards understanding developmental paradigms. Specifically, hESC-derived hematopoietic subsets could theoretically be used for a variety of therapeutic purposes such as replenishment of lymphocyte deficiency due to chemotherapy, suppression of autoimmunity by regulatory T cells, or T cell mediated anti-tumor therapy. However, we first need to establish a robust and repeatable protocol for *in vitro* differentiation.

Differences in lineage potential among independently derived hESC lines has been noted for a number of downstream target cell types and at different stages of development. In addition to gene expression heterogeneity among the hESC lines themselves, lineage skewing among hESC lines has been identified as early as commitment to the three germ layers [1]–[6]. In other reports, lineage bias between hESC lines is detected at the latest stages of development—definitive differentiation of forebrain versus hindbrain neurons, for example [7]. For the hematopoietic lineage, the potential of hESCs to develop into blood lineage cells has primarily been addressed with a restricted number of stem cell lines and differentiation methods. Several groups have reported success in generating erythrocytes, various myeloid lineage cells, B cells, and NK cells from hESCs, albeit differentiation of B cells was based primarily on expression of lineage markers rather than functional assays [8]–[17]. However, generation of T lymphocytes from the same hESC lines has been difficult to achieve, despite the fact that mouse ESCs can be easily induced to differentiate toward the T cell lineage by co-culturing with Notch-1 ligand expressing stromal cells [18]. One group has verified T lineage potential from the H1 hESC line through *in vivo* passage of hESC-derived hematopoietic progenitor cells in a humanized mouse model [19], [20]. Recently, another group reported generation of T cells from what they refer to as “hematopoietic zones” *in vitro* [21]. This is currently the sole successful report of *in vitro* T cell differentiation. However, under similar conditions, another group reported a strong lineage bias against the development of T lineage cells from hESCs, and rather an NK lineage pre-disposition [15]. These discrepancies in T lineage differentiation potential between labs using similar protocols, and the low efficiency of T cell development in successful labs highlights a need for improved understanding of hESC culture conditions and differentiation protocols before becoming clinically useful.

The basis for these differences in lineage potential among hESC lines are not completely understood but could stem from a number of variables including, but not limited to, genetic background, the quality and stage of the embryo at derivation, and the hESC isolation method. In addition, the sensitivity of hESC lines to experimental variability make it extremely difficult to compare the differentiation potential of hESC lines indirectly via published results.

Here, we set out to establish the hematopoietic and lymphoid potential of a sampling of hESC lines from various sources under different culture conditions and differentiation protocols in a side-by-side comparison at different stages of differentiation. We found significant differences in hematopoietic potential among independent hESC lines, differences in blood lineage development under different passage conditions regardless of karyotypic abnormalities, and disparities under unique directed differentiation protocols. These lineage biases were identified early in hematopoietic development and also at subsequent stages of lymphoid development. In contrast, *ex vivo* hematopoietic progenitors developed consistently and efficiently into lymphoid cells, specifically the T cell lineage, under the same *in vitro* differentiation conditions.

## Results

We sought to compare the hematopoietic potential of several hESC lines from different sources. In this analysis we included one human ES cell line reportedly skewed toward mesoderm (HuES8), one toward endoderm (HuES14), one not described (HuES15), the two lines most prevalently used by others for hESC-hematopoietic differentiation, H1 and H9, and another independently-derived hESC cell line, HSF-6 [4], [22], [23].

**Figure 1:**
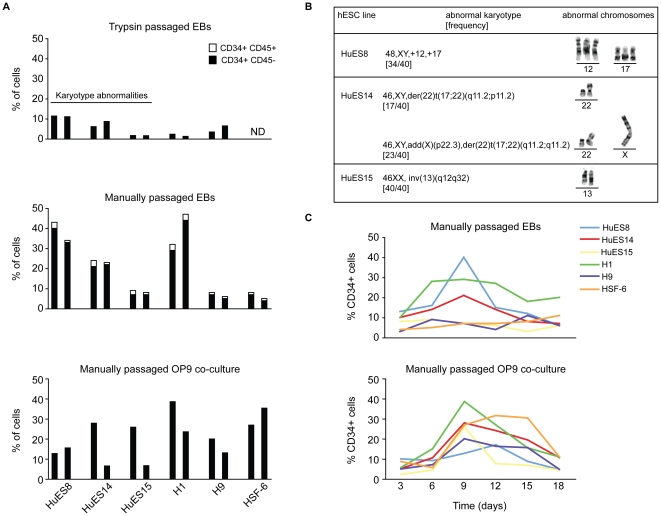
Comparative analysis of hemangioblast development from independently-derived hESC lines. Several hESC lines were differentiated as embryoid bodies (EB) for nine days after several trypsin passages (A, top panel) or after several manual passages (A, middle panel) in EB media without lineage-skewing cytokines. CD34^+^ and CD34^+^CD45^+^ development was determined by flow cytometric analysis of several cell surface markers indicative of differentiation state. The proportion of hESC-derived CD34^+^CD45^−^ cells is presented on differentiating hESCs in black. The proportion of CD34^+^CD45^+^ progenitors is indicated in white. HuES8, HuES14, and HuES15 cell lines were highly susceptible to gross karyotypic abnormalities during trypsin passage (as indicated). H1, H9, and HSF6 manually passaged cells had previously been passaged with trypsin (>5 manual passages before differentiation). (A, bottom panel) Independently-derived hESC were differentiated on an OP9 monolayer for nine days, and CD34 and CD45 cell surface expression analyzed by flow cytometry. Two representative experiments of each condition are presented. (B) Abnormal karyotypes observed in trypsin passaged cells. (C) Representative time course of CD34 expression on manually passaged, independently-derived hESCs differentiated as EBs or on an OP9 monolayer. CD34 expression was analyzed on days 3, 6, 9, 12, 15, 18.

First, we analyzed the proportion of each hESC line that gave rise to putative hemangioblasts (CD34^+^CD45^−^) and hematopoietic progenitor cells (CD34^+^CD45^+^) under various passage and differentiation parameters (Fig. 1). We compared the affect of enzymatic (trypsin treatment) versus manual passage on hematopoietic development. To assess the initial commitment to the hematopoietic lineage, we allowed hESCs to differentiate into embryoid bodies (EB) or co-cultured hESCs on an OP9 mouse bone marrow stromal cell monolayer in the absence of lineage skewing cytokines. Consistently, and regardless of cell line, manual passage gave rise to a higher proportion of hESCs differentiating to CD34^+^ cells in EB culture (Fig. 1A). Under the same differentiation conditions, enzymatically passaged hESCs also failed to up-regulate CD45, a marker indicative of hematopoietic commitment. In contrast, under the same differentiation conditions, CD45 was detectable on all the hESC lines maintained through manual passage (Fig. 1A). It has been shown that enzymatic passage of hESCs can lead to an increased frequency of karyotype abnormalities [6], [24]. Therefore, we also performed karyotype analysis periodically to determine gross karyotypic changes under different culture conditions. Trypsin-passaged HuES8, HuES14, and HuES15 hESC cultures consistently displayed gross karyotype abnormalities (Fig. 1B). However, no significant karyotype abnormalities were observed in H1 or H9 trypsin-passaged hESC cultures, nor were any chromosomal abnormalities noted in any of the manually passaged cultures. All hESC lines used throughout the manuscript maintained normal karyotypes with the exception of the top panel of Figure 1A, as noted.

We next analyzed the propensity of manually passaged hESC lines to generate CD34^+^ and CD34^+^CD45^+^ using a complementary differentiation system. Undifferentiated hESCs were harvested and plated on a monolayer of OP9 mouse bone marrow stromal cells capable of promoting hematopoietic development. Again, CD34 and CD45 expression were monitored by flow cytometry. Overall, all hESC lines consistently gave rise to CD34-expressing cells. However, several differences in the differentiation potential were noted using the two different protocols. Cell-surface CD45 was not detected at any time point on hESCs differentiated in the OP9 co-culture system (Fig. 1A, bottom panel and data not shown). In addition, although the H1 line consistently had a higher proportion of CD34^+^ cells in both differentiation conditions, other hESC lines, specifically H9 and HSF6 generated proportionally more CD34^+^ cells in the OP9 co-culture system as compared with the EB condition. We also observed that the kinetics of cell-surface CD34 expression differed significantly between hESC lines and the differentiation protocols (Fig. 1C).

**Figure 2:**
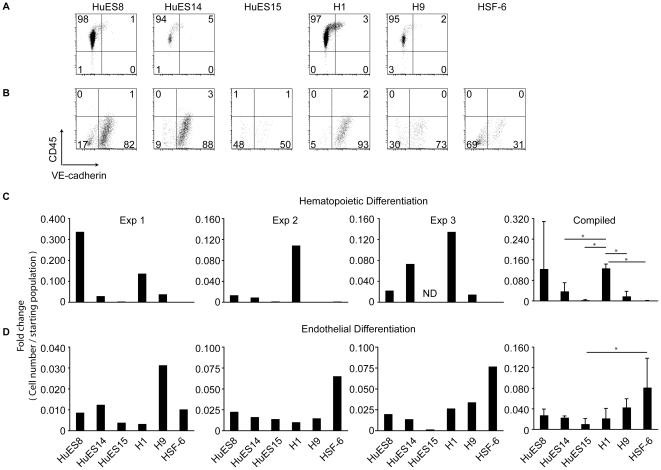
Skewed hematopoietic vs. endothelial potential from EB-derived CD34^+^CD45^−^ cells. (A) The indicated hESC lines were first differentiated as EBs in EB media without lineage skewing cytokines. CD34^+^CD45^−^ cells were enriched by fluorescence activated cell sorting (FACS) on day 10 of EB culture and differentiated on fibronectin-coated plates in the presence of IL-3, IL-6, SCF, G-CSF, Flt3L, and BMP4 for an additional 7 days. Representative flow cytometry plots of CD45 (hematopoietic marker) and VE-cadherin (endothelial marker) are presented. (B) Comparative analysis of endothelial potential from independently-derived hESC lines. Endothelial differentiation of hESC lines was determined by a two-step culture. hESCs were initially differentiated for 9 days as EBs as in (A), and CD34^+^ cells enriched by FACS. CD34^+^ cells were plated on fibronectin-coated plates in the presence of an endothelial growth factor cocktail containing bovine pituitary extract, heparin, and hVEGF, and analyzed after 7 additional days in culture. Representative flow cytometry plots of CD45 and VE-cadherin are presented. (C) Graphs depict the relative number of hematopoietic (CD45^+^) or (D) endothelial lineage (VE-cadherin^+^) cells as identified by flow cytometry over the starting (CD34^+^, CD45^−^, VE-cadherin^−^) population. Three independent experiments are shown in (C) and (D). The right panels denote the average of the three independent data sets with error bars and standard deviation between hESC lines. * denotes p<0.05.

The CD34^+^ population contains developmental intermediates capable of giving rise to multiple lineages. To compare the potential of CD34^+^ cells derived from independent hESC lines, CD34^+^CD45^−^ cells from EB cultures were enriched by fluorescence activated cell sorting (FACS) and placed into culture with differentiation media containing lineage-promoting cytokines and growth factors that provide hematopoietic- or endothelial-skewing conditions. As expected, under hematopoietic skewing conditions, CD34^+^ cells differentiated into CD45^+^VE-cadherin^−^ cells (Fig. 2A), whereas under endothelial skewing conditions, CD34^+^ cells up-regulated cell-surface expression of VE-cadherin (Fig. 2B) [25]. Of the lines generating CD45^+^ cells from CD34^+^CD45^−^ populations (HuES8, HuES14, H1, and H9), the proportion of CD45^+^ cells is comparable (Fig. 2A.) Whether generation of CD45^+^ cells in these conditions is due to loss of non-hematopoietic committed cells remains to be seen. We also noticed that the number of CD45^+^ cells relative to the starting population varied among hESC lines and between experiments (Fig. 2C). HuES8, in particular, showed extensive variability in the generation of CD45^+^ cells between experiments (Fig. 2C). This is in contrast to the ability of CD34^+^ cells from different hESCs to give rise to endothelial cells, which was more consistent from line to line (Fig. 2D). Interestingly, H1-derived CD34^+^ cells generated relatively more CD45^+^ cells while HSF-6-derived CD34^+^ cells consistently gave rise to relatively more VE-cadherin^+^ cells as compared to other hESC lines (Figs. 2C and 2D).

**Figure 3:**
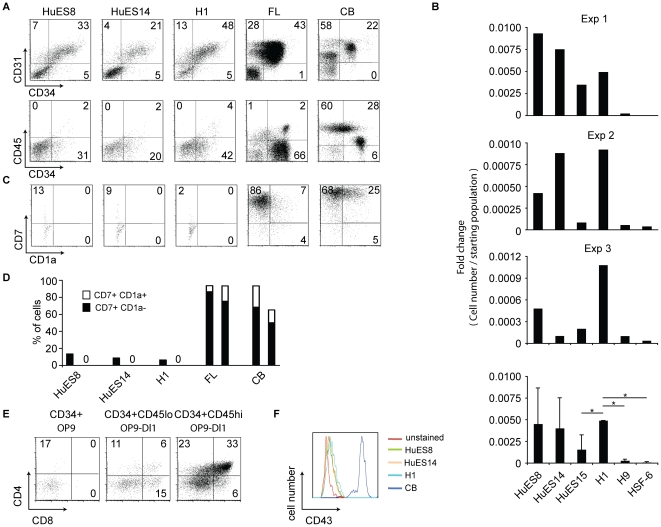
hESC-derived hematopoietic progenitor cells are phenotypically and developmentally distinct from *in vivo* hematopoietic precursors. (A) Significant phenotypic differences among hESC-derived, cord blood, and fetal liver hematopoietic progenitor cells. Representative plots of cell surface expression of CD34, CD31, and CD45 on differentiated hESCs (EB culture for 9 days), and CD34^+^ cell enriched cord blood and fetal liver. (B) Relative number of lymphocyte committed cells (as evidenced by expression CD7) as compared to input population (CD34^+^ population enriched by FACS from day 9 EBs.) Three independent experiments are shown (Exp 1–3). The bottom panel denotes the average of the three independent data sets (Exp 1–3), after normalization to the fold change of the H1 sample in each experiment. Standard deviations between hESC cells with p<0.05 (*) are also indicated. (C) Analysis of CD7 and CD1a expression from FACS enriched EB-derived, fetal liver, or cord blood CD34^+^ cells differentiated on OP9-hDL1 co-cultures for 14 days. (D) The proportion of T lineage-committed cells was determined by flow cytometric analysis of CD7 (lymphocyte) and CD1a (T cell) expression. Duplicate experiments are shown as representative of differentiation of CD34^+^ cells into CD7^+^ cells in OP9-hDL1 co-culture conditions (Enriched CD34^+^ cells from HuES15, H9, and HSF6 hESC lines failed to expand/survive in co-culture with OP9-hDL1 and are thus not presented). (E) Representative flow plots of cell-surface CD4 and CD8 expression from fetal liver CD34^+^ subsets differentiated on OP9 (left) or OP9-hDL1 (center and right) cells for 28 days. Fetal liver cells expressing CD34 were sorted to >95% purity. Additionally, fetal liver subsets were also sorted on CD34^+^CD45^lo^ or CD34^+^CD45^hi^ populations due to a gradient of CD45 expression. Cells were co-cultured on either OP9 or OP9-DL1 as indicated. (F) Flow cytometric analysis of cell-surface CD43 staining on HuES8, HuES14, and H1-derived hematopoietic progenitors (EB culture, day 9) and human cord blood progenitors. Flow plots were gated on CD34^+^ cells.

It has been shown that hESC-derived hematopoietic progenitor cells differ phenotypically from their *in vivo* fetal liver or cord blood counterparts that can easily differentiate into all hematopoietic lineages, being more similar to primitive blood cell progenitors [15], [26]–[28]. This difference might be one reason for their inability to efficiently generate all the blood lineages *in vitro*. To examine the possible differences among hematopoietic progenitors, we assessed the cell-surface expression of a cohort of markers indicative of hematopoietic differentiation state and maturity. Based on CD34, CD31, and CD45 expression, hESC-derived cells were more similar to CD34^+^ human fetal liver cells, whereas the majority of cord blood CD34^+^ cells expressed CD45^+^ cells (Fig. 3A). Since the fetal liver and cord blood CD34^+^ cells have similar lymphoid lineage differentiation potential, and the fetal liver CD34+ cells resemble hESC-derived CD34+ cells, these markers alone cannot distinguish the *in vitro* differentiation capacity of CD34^+^ cells.

Co-culture of hematopoietic progenitors on the mouse bone marrow stromal cell line, OP9, is known to support lymphocyte differentiation from a number of human hematopoietic progenitor populations [29]. Therefore, we followed the differentiation steps of hESC-derived CD34^+^ cells on an OP9 monolayer by analyzing the expression of a lymphocyte commitment marker, CD7, by flow cytometry. As shown in Figure 3B, lines HuES8, HuES14, HuES15, and H1 gave rise to a small population of CD7^+^ cells. In contrast, H9 or HSF6-derived CD34+ cells did not produce appreciable CD7^+^ cells in OP9 co-culture, and overall, lymphoid progenitor yield was low among lines (Fig. 3B). In addition, we analyzed the ability of hESC-derived CD34^+^ cells to differentiate into T cells. To test T lineage differentiation, we co-cultured hESC-derived CD34^+^ cells on OP9 stromal cells that express human delta-like 1 Notch ligand (OP9-DL1). This system has been shown to support T lineage differentiation from a variety of mouse and human progenitor cell sources [18], [29], [30]. Both human fetal liver and cord blood CD34^+^ cells generate a significant populations of cells co-expressing CD7 and CD1a marking T lineage commitment within 14 days of co-culture initiation (Figs. 3C and 3D). In contrast, no CD7^+^CD1a^+^ T cell progenitors were seen in cultures with hESC-derived CD34^+^ cells (Figs. 3C and 3D), despite the ability of the same co-culture system to support further differentiation of CD34^+^CD45^lo^ and CD34^+^CD45^hi^ fetal liver progenitors to CD4^+^CD8^+^-expressing T lineage cells (Fig. 3E).

Several groups have attempted similar differentiation of T lineage cells from hESC-derived progenitor cells with limited success *in vitro*. The only exception was a recent report that purportedly found a CD34^+^CD43^+^ population in a structurally distinct “hematopoietic zone”, which can be differentiated into CD4^+^CD8^+^ T cells by co-culturing with OP9-DL1 cells [21]. Despite extensive search under microscopes, however, we could not detect any “hematopoietic zones” as described in our hESC/OP9-DL1 co-cultures. We also analyzed expression of CD43 in CD34^+^ cells differentiating in the presence of OP9-DL1 cells. In contrast to cord blood CD34^+^ cells that did generate T lineage cells and express CD43, no expression of CD43 was detected by flow cytometry on differentiating hESC-derived CD34^+^ cells (Fig. 3F).

## Discussion

Successful development of directed differentiation protocols for all hematopoietic lineages from hESC lines would allow not only the possibility of generating blood cell subsets for therapeutic purposes, but would also permit research into early human blood cell development that is otherwise inaccessible to observe and manipulate. Despite the intense interest and investment in developing blood cell therapies from hESCs, we still lack adequate understanding of how culture conditions and differentiation protocols may affect lineage development. Here we present a comparison of the developmental potential of six independent hESC lines maintained and differentiated under multiple parameters.

Unlike previous studies that compare multiple cell culture conditions for one hESC line, or one differentiation protocol for multiple hESC lines, our approach was to directly compare multiple hESC lines under several culture and differentiation conditions [4], [31]–[34]. This provides a comprehensive side-by-side analysis of important variables on *in vitro* blood cell development. Though we do observe similar lineage potential differences in some previously compared hESC lines (HuES8, HuES14, and HuES15), we note additional differences among these lines based on passage conditions and differentiation method. First, there were significant developmental differences among the same hESC lines when passaged under different conditions. Enzymatic passage of hESCs has been shown to favor karyotype instability [6], [24], and as expected, three of the hESC lines displayed gross karyotype abnormalities following trypsin passage. Interestingly, H1 and H9 did not exhibit any karyotypic abnormalities under enzymatic passages. However, the differentiation potential of the enzymatically passaged H1 and H9 cultures as compared to manually passed H1 and H9 hESCs was dramatically impaired. These data highlight the need for manual passage of all hESC cell work.

We noted that differentiation into hematopoietic lineages varies between hESC lines and culture conditions. This is in contrast to endothelial lineage development, which was more similar between lines and experiments. This might be attributed to the endothelial lineage being a “default” pathway during lineage commitment. Using CD45 as a marker of early hematopoietic commitment, we found EB-based culture conditions superior to OP9 co-culture methods in all the hESC lines. Some of the hESC lines (HuES 8, 14, 15 and H1) consistently produced more CD34^+^CD45^+^ cells. The hESC line HuES8, for example, could produce up to 40% CD34^+^ cells within 10 days. Under hematopoietic skewing conditions, CD34^+^CD45^−^ cells from EB cultures could differentiate into CD45^+^ cells. Again, in contrast to the ability of CD34^+^ cells from all hESC lines tested to develop into endothelial cells, only three hESC lines (HuES 8, 14 and H1) consistently differentiated into the CD45^+^ early hematopoietic progenitors. A similar finding was observed when EB-derived CD34^+^ cells were co-cultured with OP9 cells, and the lymphoid marker, CD7, was used to measure differentiation into the lymphoid lineages.

We were particularly interested in improving the efficiency of generating T cells from hESCs by co-culturing CD34^+^ cells with Notch-ligand expressing OP9-DL1 cells. However, despite evidence from one group that reported development of T lineage cells from hematopoietic zones in this OP9 co-culture system, we and others have not been successful in doing so [15], [21]. The reasons behind this are not clear. The comparatively high expression of *Id* factors in hESCs and hESC-derived hematopoietic cells as compared to cord blood hematopoietic progenitors has been noted. *Id* genes antagonize T lineage development and may be one of the hurdles to *in vitro* T cell generation from hESC lines [15]. An additional test of T lineage potential may be passage of hESC-derived hematopoietic progenitors through a humanized mouse model. H1 hESC cells were shown to be capable of differentiating into T cells *in vivo* [19], [20]. However, we showed that this line under multiple conditions, nevertheless, was unable to differentiate into T cells *in vitro*.

The difficulty of differentiating hESCs into T cells is in contrast to the ease in which mouse ESCs develop into CD4^+^CD8^+^ T cells in the presence of OP9-DL1 cells. It has been suggested recently that the majority of hESCs in existence share more similarities to mouse epiblast cells than mouse ESCs derived from the blastocyst inner cell mass. Mouse epiblast stem cells are not truly pluripotent and are characterized by flattened morphology and the inability to grow from a single cell. Like hESCs, they also differentiate into teratomas. Thus, it is possible that generating new hESC lines that are more similar to mouse ESCs might be more conducive for T cell differentiation. To that end, recent studies isolating hESCs under controlled oxygen conditions or pushing existing “epiblast-like” hESCs back to a more pluripotent state by manipulation of the KLF transcriptional circuitry may provide more consistent stability of hematopoietic and T cell differentiation [35], [36]. Rigorous comparison of independent lines derived under these conditions will be needed to determine if the more “primitive” hESC lines may present better starting material for robust and repeatable hESC differentiation *in vitro*.

## Materials and Methods

### Ethics statement

This research was reviewed and approved by the UC Berkeley Stem Cell Research Oversight Committee.

### hESC Cell Culture

All hESCs have been described previously [22], [23] (see also, http://stemcells.nih.gov/research/registry/ucsf.asp). hESCs were maintained on irradiated mouse embryonic feeder cells derived from C57BL/6 mice (E12.5–E13.5) in knockout-DMEM media as described [4] (Invitrogen). hESCs were passaged by either enzymatic passage using 0.05% trypsin or manual passage using StemPro EZPassage (Invitrogen) and split at a ratio between 1∶3 to 1∶6 [4]. hESC samples were split at various time points for karyotype analysis at the Children's Hospital Oakland Research Institute.

### Differentiation protocols

Embryonic bodies (EBs) from hESCs were formed essentially as described [4]. hESC colonies were dissociated by adding 1 mg/mL collagenase IV for 10 minutes at 37°C. Plates were subsequently washed with PBS and EB media was added (no cytokines or growth factors). Dissociated colonies were removed from plates using a cell scraper and transferred into six-well low-attachment plates (Corning). Half media changes were done every other day for the duration of EB culture. EBs were dissociated for flow cytometric analysis or CD34^+^ cell enrichment by FACS by the addition of 1 mg/mL collagenase B (Roche) for 2 hours at 37°C, following by vigorous pipetting.

For hematopoietic and endothelial two-step cultures, 24 or 48 well plates were coated with fibronectin, and sorted, day 9 EB-derived CD34^+^ cells were plated in differentiation media containing IL-3, IL-6, SCF, G-CSF, Flt3L (PeproTech), and BMP4 (R&D) (hematopoietic differentiation conditions) or in Medium-199 with 20% fetal bovine serum (FBS), bovine pituitary extract (Invitrogen), heparin (Leo Pharma Inc), and hVEGF (R&D) (endothelial differentiation conditions) for 7 days with media changes on days 2, 4, and 6 as described [25], [37].

For co-culture experiments, hESC cells or FACS enriched EB-derived CD34^+^ cells were plated on OP9 or OP9-DL1 (gift from JC Zúñiga-Pflücker, University of Toronto) cells in MEM alpha media with 20% defined FBS as described [16], [18], [21]. Cultures were maintained with half media changes every other day up to 18 days. Differentiated cells were liberated by either vigorous pipetting (FACS enriched CD34^+^ cells) or by collagenase IV treatment at 37°C for 30 minutes followed by a 15 minute incubation at 37°C with 0.05% trypsin (hESC cultures).

### Flow cytometry and cell sorting

CD34^+^ cells were initially enriched from human fetal liver (ABR Inc., Alameda, CA, USA) or human cord blood (NDRI, Philadelphia, PA, USA) using the EasySep Human CD34 Positive Selection Kit (Stem Cell Technologies, Vancouver, Canada) for cell surface marker analysis by flow cytometry. Single cell suspensions of CD34-enriched fetal liver and cord blood, or dissociated EB were incubated with fluorochrome conjugated anti-human CD34 and anti-human CD45 antibodies as indicated, and washed. Cell suspensions were sorted using a Cytopeia INFLUX Sorter (BD). For flow cytometry, single cell suspensions were stained with fluorochrome conjugated anti-human CD34, CD45, VE-cadherin, CD31, CD7, CD1a, CD4, CD8 and CD43 (R&D and eBioscience). Sample acquisitions were performed on the Beckman Coulter Cytomics FC 500 or EPICS XL flow cytometer (Miami, FL, USA), and data were analyzed with FlowJo software (Tree Star, Ashland, OR, USA).
